# A 5G V2X Ecosystem Providing Internet of Vehicles †

**DOI:** 10.3390/s19030550

**Published:** 2019-01-29

**Authors:** Carlos Renato Storck, Fátima Duarte-Figueiredo

**Affiliations:** 1 Department of Electro-Electronics and Computing, Federal Center for Technological Education of Minas Gerais, Contagem 32146-054, Brazil; 2 Department of Computer Science, Pontifical Catholic University of Minas Gerais, Belo Horizonte 30535-610, Brazil; fatimafig@pucminas.br

**Keywords:** IoV, SDIoV, SDN-based 5G, V2X, Internet-based video, eMBB, mmWave communications

## Abstract

The Fifth Generation (5G) cellular network can be considered the way to the ubiquitous Internet and pervasive paradigm.The Internet of Vehicles (IoV) uses the network infrastructure to allow cars to be connected to new radio technologies, and can be supported by 5G networks. In this way, the Vehicle-to-Everything (V2X) integration needs 5G connections unavoidably. This paper presents a 5G V2X ecosystem to provide IoV. The proposed ecosystem is based on the Software-Defined Networking (SDN) concept. Considering vehicles as entertainment consumer points, the network infrastructure must be huge enough to guarantee delivery and quality. For this purpose, this paper evaluates vehicular Internet-based video services traffic and Vehicle-to-Vehicle (V2V) communications in urban and rural scenarios. Simulations were performed through the Network Simulator ns-3, employing millimeter Wave (mmWave) communications. Three metrics, data transfer rate, transmission delay, and Packet Delivery Ratio (PDR), were analyzed and compared for rural and urban IoV scenarios. The results have shown satisfactory performance to the IoV communications requirements when adopting the 5G network with V2X communications.

## 1. Introduction

Vehicular communications require some wireless communication infrastructure to always have signal coverage. The 5G cellular network emerges as a new strong alternative to allow such connections, in a reliable, secure, and fast way, providing the IoV, as well as the V2X scenarios integration. It is expected that the 5G network can be able to attend the requirements for the future IoV applications and to offer Intelligent Transport Systems (ITS) in several scenarios involving high mobility, dynamic network topology, and high data volume [1].

**Figure 1:**
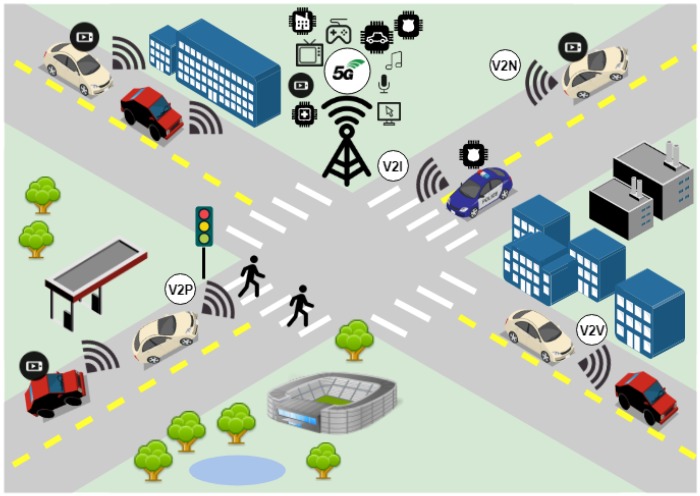
IoV through cellular network.

IoV can be considered part of intelligent cities and it is characterized as an open and integrated network system, composed by several components, including vehicles, people, and things [2], as Figure 1 illustrates. It shows that there are four V2X application types divided into this two basic operations: Device-to-Device (D2D) involving Vehicle-to-Vehicle (V2V), Vehicle-to-Infrastructure (V2I), and Vehicle-to-Pedestrian (V2P); and Vehicle-to-Network (V2N) with evolved packet switching communications [3]. In this ecosystem, safety enhancement Vehicle-to-Everything (eV2X) services or scenarios include automated driving, vehicle platooning, extended sensors, and remote driving. However, mobile entertainment with high data rate is an example of non-safety eV2X scenarios.

Among the IoV premises, there is a need for efficient management, control, and operationability, besides high capacity and assurance. In the literature, the OpenFlow (OF) protocol was indicated for the Software-Defined Internet-of-Vehicles (SDIoV) architecture as the communication protocol between the control plane and the data plane.

This paper presents an SDN-based 5G network to support IoV communications. Nowadays, with the accelerating and fostering goals of the 5G development all around the world, new architecture design, testing, and evaluation are real and urgent needs. The high complexity expected from a 5G V2X network architecture together with the imposed challenges for its development and evaluation is this work motivation. The multimedia service delivery, that is a typical non-safety eV2X application, is analyzed. The onboard entertainment services can be managed through an SDN controller and the processing can be performed on a V2X-Server. Then, an evaluation is performed of the packages’ delivery rate based on 5G V2V communication scenarios regarding the safety eV2X applications; for example, a collision avoidance system through IoV. A set of vehicle communication simulations with the 5G network, using millimeter wave links, is performed using the ns-3 simulator. As simulation results, the system throughput, the transmission delay, and packet delivery rate are analyzed with different vehicle densities.

The main contributions of this paper are the following:

**-**  The 5G SDN-based ecosystem as a basic IoV infrastructure providing multimedia service.
**-**  The simulation of rural and urban scenarios.
**-**  Results comparing urban and rural scenarios.
**-**  Results showing the proposal effectiveness.
**-**  Results comparing this proposal with others.

The remainder of this paper is organized as follows: Section 2 presents the related works; Section 3 describes relevant definitions and explanations about and for the 5G ecosystem; Section 4 describes the proposed 5G V2X ecosystem; Section 5 presents the adopted methodology; Section 6 discusses the results; and Section 7 concludes the paper.

## 2. Related Work

IoV provides all Internet services to drivers, passengers and vehicles, and the SDIoV is nothing else than the integration of SDN with IoV. This brings the need for innovation to support IoV communications, services, and applications. In this proposal, challenges are pointed out, such as high efficiency in resources use, capacity increase, management, and control in a scalable and flexible way, as well the QoS (Quality of Service) in vehicular communication networks [4].

When SDN is applied in IoV by disassociating the control and data planes, the controller simply manages the network and can shape the data traffic for the specific application. That is the reason why the SDIoV is expected to deal with the traditional vehicular networks shortcomings, offering efficient data transmission and traffic shaping in different vehicular scenarios, even with the routing protocols unaddressed in such environment limitations. In SDIoV, the packet routing can make IoV applications feasible while connected vehicles rely on the messages received from other vehicles and/or Road Side Unit (RSU) and, in such way, as the centralized controller has a global view of the whole network, an optimal route selection is easier [5].

IoV needs non-stop technology evolution to answer all the new safety requirements and use cases that come with the Cellular Vehicle-to-Everything (C-V2X) development, in reason to provide a higher performance radio, while reusing upper layers defined by the automotive industry. The Third Generation Partnership Project (3GPP) is used to define C-V2X and to specify service requirements for V2X systems, with an expectation to support several advanced scenarios [6,7].

In this direction, ITS and future vehicular networks require stringent requirements, and as already noted, these are hardly met through the IEEE 802.11p technology [8], that has 27 Mbps maximum throughput with V2X applications. As an alternative, the Long-Term Evolution (LTE) technology has been employed to support vehicle applications [9,10], however the performance is affected by interference as shown by [11].

The first V2X specifications involving cellular networks was provided in Release 14 of Long-Term Evolution-Advanced (LTE-A), focusing mainly on the aspects of vehicular safety applications, where was defined that vehicles should use a new PC5 interface for communication. It is because the LTE for vehicle standard supports side link or V2V communications using a direct interface on modes 3 and 4 which got physical layer changes introduced by Release 14, and its evolutions are being discussed on Release 15 to support 5G-V2X autonomous vehicles’ applications [12].

The 5G network integrated with SDN and Mobile Edge Computing (MEC) [13] appear as a good candidate technology for Vehicular Ad hoc Networks (VANETs) operational improvement, in terms of data rate, coverage, and QoS. There are other works that present interesting points to be considered for the 5G development common work, such as a four-tier architecture for urban traffic management [14]. Even SDN has already been used for vehicular networks, it has limitations in RSU when there is a high density of vehicles, by presents frequent handovers, what degrades its performance [15].

In [1], the authors found that there is a minimum transmission delay, in accordance with different densities of vehicles. With the fog cell use, the authors were able to demonstrate an improvement on the transport management system performance. However, problems on the multi-hop routing retransmission and gateway vehicle selection on the proposed structure persist, which directly affects the system performance. Finally, a next generation 5G topology structure, using SDN controllers and a fog computing framework with zones and clusters to avoid frequent handovers between vehicles and RSUs is presented in [16].

## 3. 5G Standards and Features

This section presents the 5G standardization and the development efforts (Section 3.1), and the features of 5G mobile communications systems (Section 3.2).

### 3.1. 5G Standardization and Development

The 5G networks specifications are being discussed and analyzed by the academy, by Standard Development Organizations (SDOs), by some consortia, by government and by industry. A panorama of institutions that contribute to the development and standardization of 5G networks is presented on Table 1, as well as the main contributions.

Among the most recent, 5G Automotive Association (5GAA) consists of automotive and telecommunications companies, among them Audi, BMW, Daimler, Ericsson, Huawei, Intel, Nokia, and Qualcomm. This organization aims to deliver mobility and transportation solutions through technical studies, testing, analysis and deployment of the C-V2X [28].

Some researches are being developed by the academy, all over the world. An example in Europe is the University of Surrey. It has the largest center of 5G technology research of the United Kingdom, called 5G Innovation Centre (5GIC), with the financing of £ 16 million from the Higher Education for England, also counting on more £ 68 million from the industry and partners [29].

In the United States, the University of New York is an example and it has an academic research center for investigation about new technologies of wireless communication, involving devices, networks and applications. The main focus is the use of mmWave over 10 GHz. With the higher frequencies through mmWave and, consequently, greater propagation and penetration losses, researchers have studied beam formation techniques in order to improve the signal level [30].

In South America, the 4th Coordinated Call among European Union–Brazil (EU-BR) was launched in Brazil, on February 2017, through the Brazilian National Research and Educational Network (RNP) and Brazilian Ministry of Science, Technology and Innovation (MCTI). It predicted one project with the 5G theme and investment of R$3.248 million (Reais), amount that proves and emphasizes the importance of this new network development. Also, MCTI and National Telecommunications Agency (ANATEL) created the 5G Brazil Project to foment the 5G ecosystem creation in Brazil [31].

All over the Asia continent, there are some 5G researches. For example, in the Malaysia Technology University, they created the Ericsson 5GIC that has signed a partnership with local and global businesses to support more 5G technology investigations [32].

### 3.2. 5G Features

The 5G mobile communications systems are considered as new disruptive technology and can be an end-to-end ecosystem that will allow a fully mobile and connected society. Examples of design aspects for 5G architectures were presented by [33,34]. The 5G networks will account on the new radio, supporting technologies such as LTE-A, mmWave, Wi-Fi and Wireless Gigabit (WiGig), serving from cells with ultra-density to D2D [35,36]. The set of required resources and functionalities to develop the 5G network is provided through reference architectures found at the 3GPP Release 15 [37] and Technical Specification (TS) 23.501 [38].

The 5G network use cases are typed by enhanced Mobile Broadband (eMBB), massive Machine-Type Communication (mMTC) and Ultra-Reliable and Low Latency Communication (URLLC) [39]. Access to multimedia content, services and data such as virtual reality and augmented reality are typical applications of eMBB use case, providing a perfect user experience. mMTC use case is characterized by the Internet of Things (IoT) and D2D applications use with large number of connected devices and typical transmission of low volume data and non-delay-sensitive, with low-cost devices and long battery life. Wireless control of industrial manufacturing or production processes, IoT applications, remote medical surgery, delivery automation in a smart grid and transportation safety are examples of URLLC use case, demanding stringent requirements for reliability, latency, and network availability.

The 5G Access Network (5G-AN) can be designed in the 5G network considering the following aspects: outdoor or indoor, wide area macro or micro coverage. The types of 5G deployment scenarios are rural, suburban, urban, dense urban and indoor. The Rural Macro (RMa) environment focuses on larger and continuous coverage, supporting high-speed vehicles. The Suburban Macro (SMa) focuses on residential areas coverage as well as on rural towns with low-rise buildings. The Urban Macro (UMa) usually has on large cells and continuous and ubiquitous coverage. Dense Urban or Urban Micro (UMi) has high traffic loads, outdoor and outdoor-to-indoor coverage. Finally, Indoor Hotspot (InH) is characterized by small coverage and high capacity.

**Figure 2:**
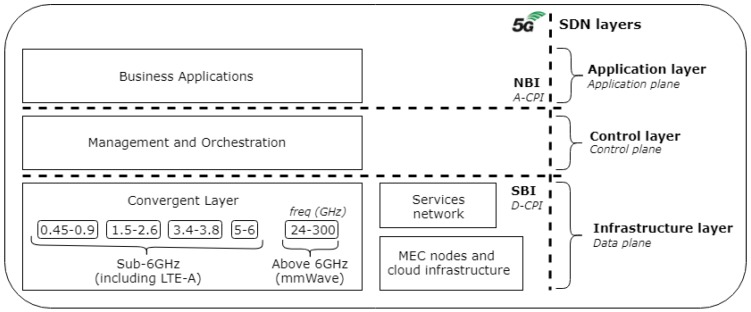
5G integration with SDN.

Considering 5G a heterogeneous high-speed network, several advanced features are expected and its development is a rather complex process. The operational network efficiency improvement will occur through technologies integration and use, such as cloud, fog, VANETs, Network Functions Virtualization (NFV) and SDN [40,41]. Figure 2 shows a 5G system integration view and its relationship with SDN. The services offered on the 5G network are the application layer. The SDN paradigm allows a network to be directly programmable by decoupling the control plane and the data plane. It puts the network intelligence on SDN controllers enabling the network dynamic management [42].

As Figure 2 shows, the business applications layer has the services and it communicates with the management and orchestration layer. Technologies such as LTE-A and mmWave with high frequencies, are part of the physical system, called convergent layer. The SDN functional architecture is composed by the Infrastructure layer represented by the data plane with the network elements, the Control layer represented by the SDN controller and, finally, the Application layer represented by the service plane with the services and the applications. Between the layers, there are the Southbound Interface (SBI) and Northbound Interface (NBI) that are communication interfaces which, respectively, pass along the characteristics of the network elements to the controller via the Data-Controller Plane Interface (D-CPI), and via the Application-Controller Plane Interface (A-CPI) connect it to the SDN application. The coordinators are responsible by the resources installation and by specific policies passed from the management layer through the Operations Support System (OSS) or Business Support System (BSS). The isolation of the user plane from the control plane by SDN is adequate to the 5G-IoV complex and heavy traffic, enabling secure and direct communication through the OpenFlow protocol.

## 4. Proposed 5G V2X Ecosystem

**Figure 3:**
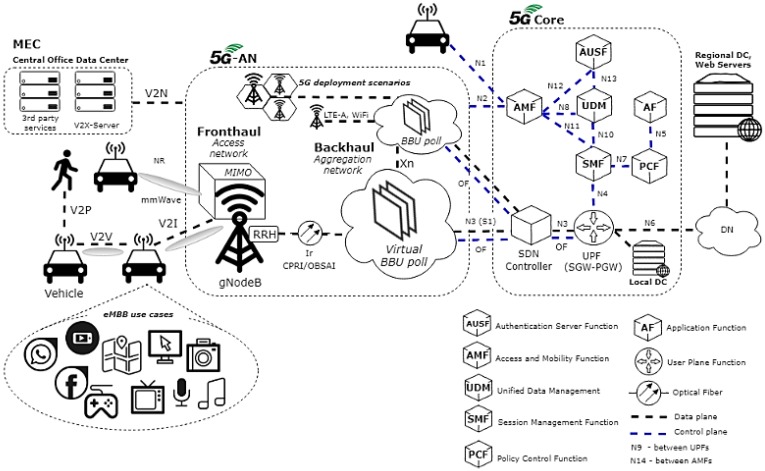
5G V2X ecosystem.

This section describes the 5G V2X ecosystem proposed architecture. The fast mobility and the intense traffic in a next-generation vehicular network have motivated this work. The proposed 5G ecosystem puts the network intelligence on the SDN controller enabling the network dynamic management. Figure 3 shows the 5G V2X ecosystem proposed. It has a connection between the Baseband Unit (BBU) polls and an SDN controller. It communicates through the backhaul with the Core Network (CN) called 5G Core Network (5GC) with the separation of the control plane from the data plane. At the CN, the Serving Gateway (S-GW) and Packet data network Gateway (P-GW) elements are unified as User Plane Function (UPF). Servers such as the V2X-Server can be enabled in the Central Office Data Center to support new services.

At the CN, the SDN controller is inserted and the OpenFlow (OF) protocol is adopted [43,44]. The OF has usually been employed and becomes a suitable alternative for 5G V2X scenarios since it enables secure and direct communication with the SDN controller. When the control plane and the data plane are separated, IP streams are routed from 5G next generation network Node Base Station (gNodeB) to UPF using the SDN controller.

On the control plane there are the reference points N1 between the vehicle and the Access and Mobility Management Function (AMF), N2 between 5G-AN and the AMF, N4 between the Session Management Function (SMF), and the UPF, N5 between the Policy Control Function (PCF) and an Application Function (AF), N7 between the SMF and the PCF, N8 between the Unified Data Management (UDM) and AMF, N10 between the UDM and the SMF, N11 between the AMF and the SMF, N12 between the AMF and the Authentication Server Function (AUSF) and N13 between the AUSF and the UDM. On the data plane are the reference points N3 between 5G-AN and the UPF, N6 between the UPF and a Data Network (DN) at the cloud and N9 between two UPFs.

To support V2X over 5G systems, the providing dedicated logical networks (i.e., slices) with virtualized functionalities over a common 5G physical infrastructure is presented in [45]. The 5G V2X network slicing view can logically isolate control plane and user plane. Such as in our work, the 5G architecture presented follows 3GPP specifications composed by network functions and reference connection points, as well the four types of V2X communication modes identified as V2V, V2P, V2I and V2N. In the work, the authors report that in the CN domain, the AMF, UDM, and the AUSF can be shared among multiple slices. The UPF and SMF network functions can be dedicated per each slice. The SDN function is the remote configuration of the physical network, ensuring the resources reservation for the various slices which are being demanded by different types of V2X services. Each vehicle may require separate slices, one for each type of service, such as one slice for autonomous driving and another for entertainment on board. The vision of a preliminary 5G network slicing architecture is presented in a three-layer model (Business layer, Service layer and Infrastructure layer) with also an additional layer for management and orchestration. In our proposal, three layers are presented based on the Figure 2, with the management and orchestration functions embedded in the control layer. The practical validation through implementation is a future work.

**Figure 4:**
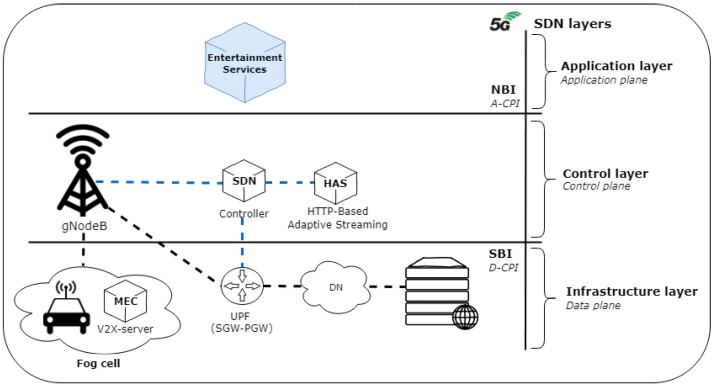
Logical view of SDIoV entertainment services.

The entertainment services delivery to people on board is one of applications of the IoV. One of the problems to be treated is how to make vehicles viable as entertainment centers, ensuring high Quality of Experience (QoE). The QoE that is perceived by the user while using the multimedia services depends significantly on the network Quality of Service (QoS) [46]. Therefore, there is a need of an architecture that supports IoV, with well-established standards and also extensible to new ones [47]. In this direction, 5G infrastructure based on software defined concepts can support IoV applications [4]. For this purpose, Figure 4 shows the logical structure for entertainment services in 5G V2X ecosystem, divided into three layers, following the concept of SDN. The infrastructure layer consists of the vehicles, UPF and other service provider elements to the users. In the control layer, the SDN controller manages gNodeB and Hypertext Transfer Protocol based (HTTP) Adaptive Streaming (HAS). The application layer represents the abstraction provided by the controller through the entertainment services delivery.

In the SDN structure, the data plane includes vehicles and gNodeBs and is responsible for collecting data. The control plane includes gNodeBs and V2X-servers and is responsible for deriving control instructions. The SDN controller must count on the data collection module based on the data plane, besides the network monitoring module. Finally, the application plane is responsible for the rules in the SDN structure.

**Figure 5:**
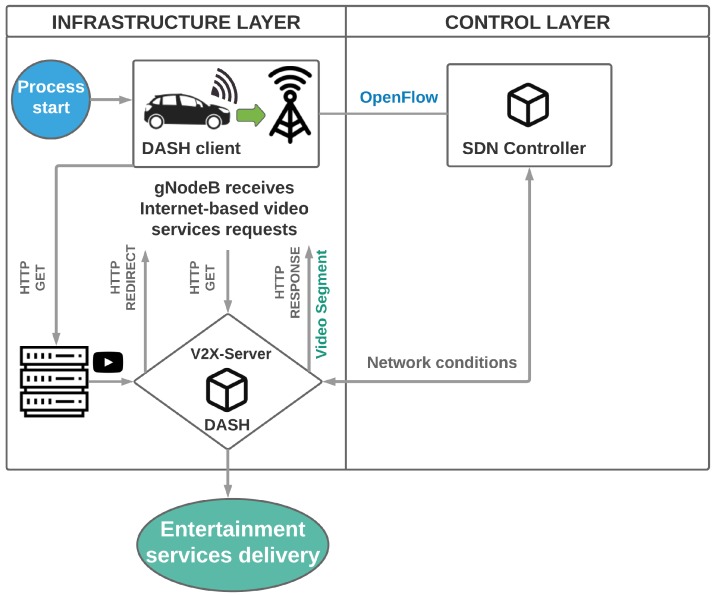
Operation of the proposed architecture.

The general scheme of the proposed architecture operation is shown in Figure 5. Initially, gNodeB receives the Internet-based video services requests from each vehicle connected to it. The network condition monitoring is performed on the controller. When the video server receives the HTTP GET, it pushes the video to the V2X-Server and notifies vehicles by sending an HTTP REDIRECT. Dynamic Adaptive Streaming over HTTP (DASH) is considered one of the most successful streaming technologies (TCP stream) for video on demand. DASH was deployed on the V2X-Server using the MEC concept, allowing a processing that is closer to the end-user. Through adaptive algorithms, DASH allows the dynamic adjustment of the video representation for the network conditions. Then, the vehicle sends another HTTP GET request for the video request to the V2X-Server, which answers by providing the appropriate video segment, after processing.

The vehicles can implement the following functions for the various V2X services: vehicle and environmental information collection module through sensors; positioning module; and communication module including V2I and V2V. The V2X association with MEC (V2X-Server that is used for HAS application) counts on a cache module to save recent and popular contents, as well as to process edge information after orchestration decisions by network function virtualization. It can also be used for the SDN application plane processing, such as security and efficiency services.

This section presented the proposal of a 5G V2X ecosystem based on TS 23.501. The proposed ecosystem supports IoV communications and entertainment services delivery.

## 5. Materials and Methods

Evaluations through experiments and simulations are crucial to the success of 5G-IoV research and development. Prototypes, simulations, and testing of 5G networks are being carried out by the academic community and many manufacturers around the world. The simulation, the modeling and the prototyping, allow the system evaluation without a real network implementation. It means faster new features, protocols and development with low costs.

### 5.1. Modeling and Simulation

Table 2 lists the main tools and available frameworks to promote the 5G technology advance. Among the platforms listed, only the last two are not proprietary. National Instruments is an American company that provides tests, measurements, and embedded systems, besides proprietary software products for solution development. For 5G networks, it offers LabVIEW Communications software that is an engineering one designed for applications that require testing, measurement, and control, with quick access to hardware and information from the data plane, and was specially developed for wireless communication system prototyping [48].

MathWorks is a manufacturer that provides software such as MATLAB (analyses data, develops algorithms, and creates mathematical models) and Simulink (runs simulations, generates code, and tests and verifies embedded systems), that can offer an integrated environment for simulating, testing and prototyping wireless technologies [49].

Fraunhofer Institute for Open Communication Systems is a German institute that develops 5G networks and SDN research through next generation network infrastructures. Commercially, it provides, for prototyping and testing achievement, a set of tools through the 5G Playground composed by the components: 1st, Open5GCore—scalable, low-delay and highly reconfigurable approach to network core; 2nd, Open Baton—NFV orchestration; 3rd, Open SDN Core—SDN and NFV resources and backhaul addressing; and 4th, Open 5GMTC—connectivity of an endless number of network devices [50].

Developed by the Riverbed organization, the old Optimum Network Performance (OPNET) open software has gained a new name: Riverbed Modeler which is a discrete event simulation platform for communication networks analyzing and designing. It has a set of protocols and technologies with a development environment, allowing the network technologies modeling. Among its advantages, cited by the manufacturer, are tests and demonstrations of technology projects before production, productivity increase, protocols and proprietary wireless technologies development and, finally, the improvement evaluation on already consolidated standards-based protocols [51].

The Open Air Interface Software Alliance (OSA) is a French organization whose open platform of experiments and prototyping is made available to the community, where strategic work areas are established, such as 5G-SDN systems and heterogeneous 5G networks [52].

Several works in the literature use the discrete-event simulator on ns-3 network [53] and the LTE-EPC Network Simulator (LENA) module [54]. It is pointed out that the ns-3 simulator has already been validated for the LTE module and has been indicated as the best choice for complex scenarios, as demonstrated by [55]. Completing for the scope of work, the Keysight Technologies is a company that provides tools for 5G signal modulation, such as the Advanced Cellular Pack for Simulation and 5G Protocol R&D Toolset [56]. The Keysight Technologies provides a set of data transfer rate tests in eMBB scenarios, through emulation. However, there are limitations in this tool, since only layers one to three are emulated. The behavior of the system is not complete since kind of scenarios and the SDN controller are not emulated. It is important to evaluate the lower levels, but the complete upper levels evaluation is also crucial to the system success.

### 5.2. The 5G Ecosystem Simulation Methodology

In order to evaluate the 5G ecosystem as the IoV infrastructure support, we have conducted the evaluation of a set of data transfer tests in enhanced Mobile Broadband (eMBB) scenarios with Internet-based video services [57]. The 5G mmWave communications were used in the simulations. Simulations were conducted through ns-3 simulator. Scenarios were modeled and codes were developed in ns-3. Some modules were modified in order to support the integration of the proposed ecosystem. V2X scenarios were simulated with different vehicle densities. The simulations were executed in a high-performance computing environment provided through cluster F37 of the CEFET-MG, a brazilian public education institution. The cluster is open to students and professors of the institution who wish to develop research and teaching projects. The F37 cluster has 32 machines of shared use, in 3 different configurations which are grouped into the small, medium and large rows. The simulations were executed in the large row (4 Supermicro machines, 64 physical threads without hyperthread, and 128 GB RAM) under QoS requirements called part2d and part10d, which have a maximum time of 2 and 10 days, respectively. This is one of the first works that compares MATLAB’s results [1,16] with evaluations of VANETs integrated to 5G. These simulations allow executing all the protocols and modeling real scenarios with an intense mobility of the expected 5G networks. Generally, simulations performed through the MATLAB present a mathematical efficiency. In contrast, they are not able to capture the complexity of the network on a much higher level of details, because it presents limitations on the network important aspects, for example, the mobility deal.

For mmWave communications, the mmWave ns-3 module was used [58,59]. On LENA module, it divides the available resources between active flows. On mmWave module, the resources separation is based on time division multiple access with the allocation of time domain symbols inside a periodic sub-frame for different users in Downlink (DL) or Uplink (UL) directions. The flow rate calculation on bits/s (bits by second) for each vehicle is given by T (throughput), where S (M, B) represents the size of the transport block as defined by 3GPP TS 36.213, M is the modulation and coding scheme, B is the transmission bandwidth configuration in number of resource blocks, and τ represents the duration of the Transmission Time Interval (TTI), formulated by the Equation (1):

(1) $$T=\frac{S(M,B)}{8\tau }$$

For video traffic, the Dynamic Adaptive Streaming over HTTP (DASH) was adopted. Three adaptive algorithms were tested: FESTIVE, PANDA, and TOBASCO2 [60]. We suppose that there is one video server, for each video required, across the network.

To support the SDN controller, the OpenFlow 1.3 (OFSwitch13) module was adopted [61]. Based on the SDN concept, the OF protocol was simulated. The structure was composed by the controller and the network OF elements [62,63]. With this module, it was possible to extend the controller application interface to implement the desired control logic to orchestrate the network, in our case, the delivery of multimedia services.

The simulations were executed assuming a complete traffic model which means that the base stations are always transmitting and receiving multimedia data, with download of data through links and mmWave devices, CN based on SDN, and 5G scenarios. The parameters adopted to model each scenario considered the node numbers for each gNodeB (numgNB), gNodeB height (hgNB) in meters, User Equipment height (hUE) in meters, maximum and minimum distance (maxdist, mindist), eMBB active vehicles (numVehicles), and vehicles speed (speed), as Table 3 shown. All users are active and communicate all the runtime.

Three deployment scenarios based on Technical Recommendation (TR) 38.913 were simulated: rural macro, urban macro and urban micro [64]. First of all, the RMa (Rural Macro) scenario supporting high-speed vehicles. The choice of this scenario is especially due to the expectancy of the cellular network traffic increase, considering the rural area with its implementation sparser than the other 5G deployment scenarios. Second, UMa (Urban Macro) is simulated to verify the behavior in urban areas. Finally, we have simulated an UMi (Urban Micro) environment.

**Figure 6:**
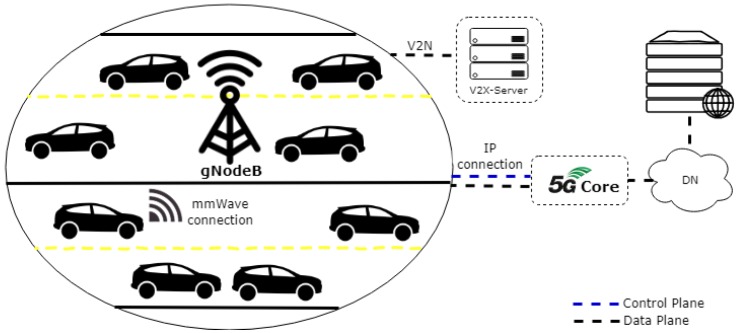
Network topology simulated of a fog cell.

The network topology simulated is presented in Figure 6. It has one gNodeB and 10 to 60 users for simulated scenarios. It was assumed one user enhanced Mobile Broadband (eMBB) by vehicle, since the European Environment Agency has defined that the average number of people in a passenger car is 1.45, as during the journey the driver consumes less data than the respective passengers [65]. The traffic model used was provided by the Simulation of Urban Mobility (SUMO) the support of ns2-mobility helper class of ns-3. As can be seen in Figure 6, four street lanes were adopted.

Each simulated vehicle was placed on one of the four street lanes. The vehicles move at constant speed in the same scenario (based on the recommendations of 3GPP TR 38.913) and in the same direction in each one of the tracks, from right to left on the 1st track (1st and 2nd lanes) and from left to right on the 2nd track (3rd and 4th lanes). Since the two tracks with the four lanes are used, each vehicle is randomly assigned to one of the lanes, being positioned following an exponentially distributed inter-vehicular spacing. Considering an expected high mobility of the 5G networks, all vehicles travel at 120 km/h, based on the rural scenario of 3GPP TR 38.913. For urban UMa and UMi scenarios, all vehicles travel at 30 km/h. Through the mmWave 3GPP Channel class, spatial consistency across the entire vehicle motion path was also enabled.

For V2V communication scenarios, the main network goal is to deliver packages for as many vehicles as possible, when it is necessary to inform the critical conditions of the road, such as accidents, road adverse conditions and abrupt braking, in order to avoid collisions between the vehicles. The network becomes reliable when the highest delivery rates are presented. The Packet Delivery Ratio (PDR) is the relation among the amount of the vehicles which receive the transmitted packets and the number of vehicles in the network, formulated by the Equation (2). In this way, based on 5G V2V communication scenarios, an evaluation was carried out, adopting rural and urban scenarios. The results are presented in the next section.

(2) $$PDR=\frac{Total\;number\;of\;vehicles\;receiving\;packages}{Total\;vehicles\;on\;the\;network}$$

Table 4 presents the adopted simulation parameters. The 3GPP mmWave channel model was used to perform the initial connection of the active vehicle to the gNodeB using mmWave communications and considering the parameters of frequency, bandwidth, number of sub-bands, channel conditions, and fading listed in Table. The Round Robin scheduling was used.

The centre carrier frequency is 28 GHz and 1 frame contains 10 sub-frames, each one of 1 ms, and contains 24 Orthogonal Frequency Division Multiplexing (OFDM) symbols with 100 μs resulting in a bandwidth of 1 GHz. Each sub-band corresponds to 13.89 MHz and contains 48 sub-carries.

In addition, Hybrid Automatic Repeat Request (HARQ) and Radio Link Control-Acknowledge Mode (RLC-AM) mechanisms were enabled. For retransmission, V2X incorporates the HARQ with the purpose of achieving greater efficiency at the link level, higher transmission range and reliability on the performance [3]. The segment size was set to 1446 bytes. On the simulation, we adopted 180 s as total time.

The video adopted in the simulations represents a real DASH encoded one that contains 442 s and 221 segments (2 s per segment) and 8 representations with variable bit rate from 0.088 to 20.5 Mbps. The size of the segments is provided in a Matrix (n, m) where [n] represents each one of the 8 representations and [m] represents each segment.

## 6. Results and Discussion

**Figure 7:**
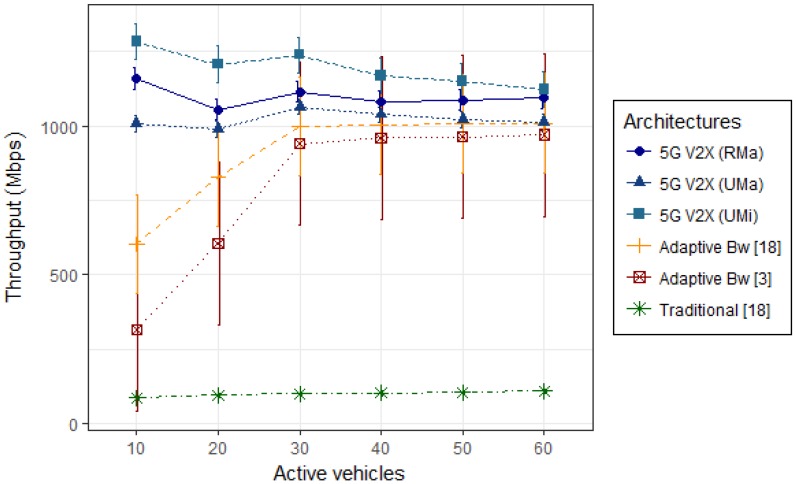
Network throughput.

**Figure 8:**
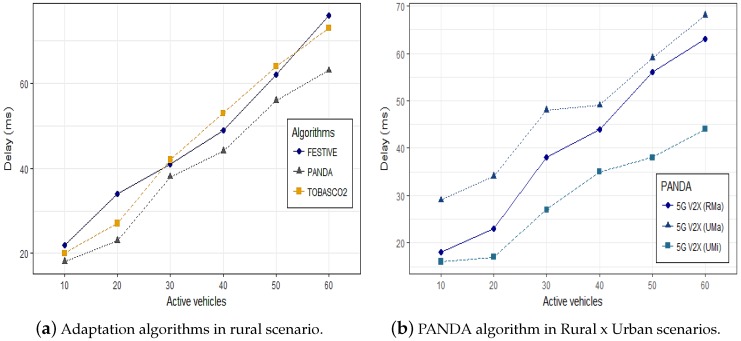
PANDA algorithm in Rural x Urban scenarios.

Video streaming applications are very sensitive to bad connections. In order to show the effectiveness of our 5G ecosystem supporting the V2X IoV connectivity, we show two important performance metrics: throughput and delay. Results obtained through simulations are presented in the Figure 7 and Figure 8 graphics, with 10, 20, 30, 40, 50, and 60 active vehicles.

Figure 7 graphic shows the obtained 5G V2X ecosystem throughput with 95% confidence interval, for 10 simulation trials using PANDA algorithm. The flow rate was obtained through the Packet Data Convergence Protocol (PDCP), which provides, for each TTI, the amount of transferred data by the users. On the 5G network, the reference value of the maximum data rate is 1–10 Gbps, which can support, in some scenarios, up to 20 Gbps. By the experiments, the data rate required by the eMBB use case was reached in scenario simulated.

As seen in Figure 7 graphic, the rural scenario performed well, considering an environment with vehicles traveling at high speed. It was observed that the urban macro scenario presented a minor throughput than the urban micro and rural scenarios [57]. This is justified by the interference of blockages by obstacles considering a larger coverage in an urban area. As expected, with low distance and vehicle mobility at 30 km/h, the urban micro scenario presented higher performance than the rural scenario. For low densities, with 10 and 20 active vehicles, the rural scenario presents better throughput, around 11.9% and 5.6%, respectively, in relation to the urban macro scenario. When comparing the rural to the urban micro scenario, the throughput achieved by the rural scenario is lower, between 9.6% to 12.5%, when 10 and 20 were simulated. The difference between the urban micro scenario and the urban macro scenario is 21.5% for the density of ten active vehicles, and 18.2% for 20 active vehicles. When simulating 30 vehicles, the percentage differences decrease between the simulated scenarios. For densities of 50 and 60 vehicles, the difference between the urban micro and macro scenarios is 11% and 9.8%, respectively. The difference from the rural scenario to the urban macro scenario is 5.6% for 50 active vehicles and 7.4% for 60 active vehicles. The difference from the urban micro scenario is 5.4% and 2.4%, respectively, when compared to the rural scenario. We can say that the urban micro is the scenario that achieved the best throughput with the 5G ecosystem.

The throughput increases as the density increases in the results obtained by [1,16] because, once the distance decreases between adjacent vehicles, the successful transmission probability increases. The throughput is maintained constant after the density of 30 vehicles. The reason is that all available bandwidth in a fog cell has already been allocated for vehicles. Using the direct communication approach, it was observed that using our 5G V2X ecosystem, the throughput does not depend on the probability of hops between vehicles, like the other literature proposals. The 5G V2X ecosystem has presented better result with any density of vehicles.

Comparing with the best results obtained by the implementation of the adaptive bandwidth allocation [1,16] and the baseline of the traditional architecture [16], it was verified that the obtained throughput in this achieved solution has gotten a better flow than the previously presented solutions, which guarantees a satisfactory QoS for streaming services in the V2X scenario. One of the factors that contributed to increase in flow is the incorporation of V2X-Server into the 5G V2X ecosystem.

Figure 8 graphic shows the average delay that reflects the spent time for users’ data transmission end-to-end in a fog cell for video transmission. The results compare the adoption of the FESTIVE, PANDA and TOBASCO2 algorithms [60] with the SDN controller in rural macro scenario in Figure 8a. Next, the execution of the PANDA algorithm by simulated scenario is depicted in Figure 8b.

The 3GPP TS 23.203 specification provides latency budgets [66]. To the video traffic requirements defined by TS 23.203, the maximum delay value is 300 ms. In [1], it claims a delay of 0.06 ms, however it is the average transmission delay, adopting the hop of vehicle communications. However, in this work, it was adopted end-to-end average delay in the 5G V2X fog cell for stream video transmission. In Figure 8a, from 30 vehicles, the values obtained by the FESTIVE and PANDA algorithms present a similar behavior, unlike the PANDA algorithm that presents a differentiation from the density with 20 vehicles. The obtained results have met the expected value, and the minimum average transmission delay of 18 ms was observed for the maximum distance of 320 m in the end-to-end fog cell 5G V2X with the PANDA algorithm, which demonstrated a better performance and stability when adopted in the proposed 5G ecosystem.

**Figure 9:**
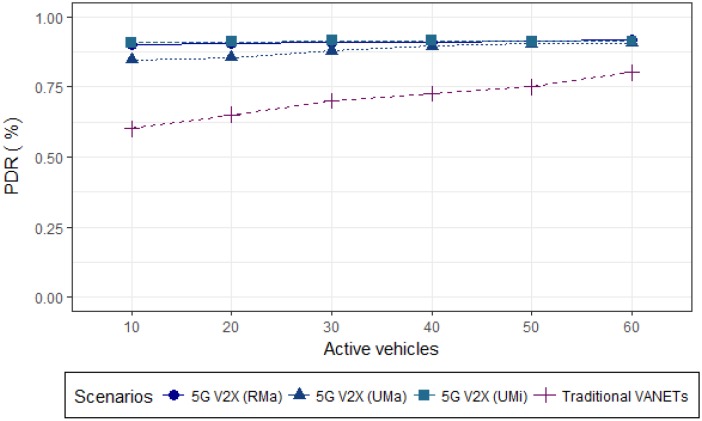
Packet Delivery Ratio in 5G V2V communications.

Figure 9 graphic shows the V2V packet delivery results. It is possible to observe, that the delivery rate depends on vehicle density.

In the literature, traditional VANETs present the PDR of between 60–80%. By simulations, when adopting 5G millimeter waves to transmit the messages, considering the confidence interval of 95%, a constant behavior is observed, between 84% and 91% of vehicles that received the package, in all vehicle densities. Generally, the delivery rate tends to be low in scenarios with low vehicle density. However, it is noted that the obtained result is due to the adoption of 5G in a cell of mist, evidencing the approach efficiency to provide IoV.

With this paper 5G V2X ecosystem proposal, the evaluation of many expected scenarios with SDIoV applications becomes possible. With an SDIoV architecture, services can be improved allowing adequate support for IoV communications, such as the tests performed with on board entertainment services. In addition, this ecosystem proposal contributes to an urgent demand for the construction of scenarios and initial 5G use cases in order to prove that the IMT-2020 standard is feasible for deployment in the short and medium term, thus justifying the investments in CAPEX and OPEX. Possible policies can be thought and projected, as well as new services for SDIoV, with the proposed ecosystem.

## 7. Conclusions

The 5G networks have several challenges to fulfill the IMT-2020 standard requirements. They must suit the new characteristics of the new network model in the operational level. The high complexity expected from a heterogeneous 5G V2X network architecture, together with the imposed challenges for improvement the multimedia services efficiency were this work motivation.

For the 5G V2X research and development success, experiments are crucial for the system evaluation and the real implementation. Huge investments are being made by academia, industry, manufacturers and governments, around the world, to the first simulations and tests of the infrastructure that will be base of the future Internet, including IoT and IoV.

This paper presented a 5G V2X ecosystem and evaluated scenarios with the eMBB use case, and V2V communications. Internet-based video services were simulated through the ecosystem with the goal to investigate it. The provision of IoV application (entertainment on board services delivery) through an SDIoV architecture has been evaluated. It was simulated through the ns-3 simulator, that is indicated as the best choice for complex scenarios and cellular systems. It was adopted in this work. The presented results show that the proposal can improve the IoV communications requirements under high-mobility (120 km/h vehicles in a rural environment) and high-density conditions (urban scenarios with good performance results).

The future will have everything connected, integrated and a fully technology convergence is expected with the 5G evolution. Previous works have used MATLAB to evaluate the radio network in 5G vehicular architectures. The present work analyzes the results using ns-3, making this study one of the firsts with practical evaluation of end-to-end 5G integrated with VANETs obtained by ns-3 and compared with the MATLAB’s ones. In this way, simulations were conducted showing as result the satisfactory performance of the 5G scenarios evaluated. The IoV services efficiency in 5G V2X networks can be reached with technologies integration. Scientific efforts are present world around to find appropriated solutions such as the SDIoV architecture that comes as a light for future directions.

With the 5G networks implantation until 2020, studies and researches about its architecture, implementation and management are urgent. The 5G V2X ecosystem was conceived as an open architecture project, enabling the integration of various technologies, enhancement and extension of new protocols. The incorporation of elements into the proposed ecosystem structure, for example SDN, V2X and MEC servers, aims to allow the creation of new mechanisms for improved network services. Evaluations of other types of services can be carried out.

In future works, V2V, V2I, and V2P simulations will be investigated using the developed ecosystem, assessing the situational information messages exchange (beacon) by the probability of situational awareness blackouts in the vehicular network. A new HAS algorithm can be created and the evaluation of other traffic models and measurements in real environments can be done.

## Figures and Tables

**Table 1:** Panorama of 5G networks development and standardization.

Institution	Projects and Initiatives	Target	Main Contributions
ITU [17]	International Mobile Telecommunications for 2020 and Beyond (IMT-2020)	Radio regulations; Operational aspects; Protocols and test specifications; Performance, QoS and QoE; Security	Recommendations (standards)
3GPP [18]	5G specifications	Radio access network; Service and systems aspects; Core network and terminals	Releases; Technical specifications
ETSI [19]	5G technologies	mmWave transmission; Next generation protocols; MEC; NFV	Technical specifications
NGMN [20]	Next Generation Mobile Networks (NGMN) 5G Initiative	Technology evolution towards 5G	White Papers
ATIS [21]	Technical forum	Incubator of new business models	White Papers
5G-PPP [22]	Working groups and various 5G Public Private Partnership (5G-PPP) projects	5G infrastructure; 5G architecture	White Papers
IEEE Future Networks [23]	Technical community	Providing practical, timely technical and theoretical content; Development and deployment of 5G	Research publications
5G Americas [24]	5G network development on Americas	Support and promote the full development of wireless technology capabilities	White Papers
5GMF [25]	5G research and development by industry	5G radio access technologies; Network technologies for 5G	5GMF White Paper
Verizon 5G TF [26]	Forum and technical specifications	Specifications for physical layer, MAC, RLC, PDCP, and RRC	5G specifications
5TONIC [27]	Open research laboratory	SDN; NFV; Physical and MAC layer	5G technologies
5GAA [28]	Mobility and transportation services	Use cases and technical requirements; System architecture; Standards and spectrum; Business models	White Papers

**Table 2:** Tools and frameworks for prototyping and simulation.

Supplier	Name	Brief Description
National Instruments	Lab VIEW Communications	Prototyping of wireless communication systems
MathWorks	MATLAB and Simulink	5G wireless system model
Fraunhofer Institute	5G Playground	Prototyping of 5G networks, including SDN
Riverbed Technologies	Riverbed Modeler	A suite of protocols and technologies to design, model, and analyze
Keysight Technologies	Advanced Cellular Pack for Simulation	Pre-5G physical layer measurements based on the Verizon 5G specifications
Open Air Interface	Open Air Interface	Software and tools for 5G wireless research
NSNAM	ns-3	Discrete-event network simulator

**Table 3:** Parameters of 5G V2X scenarios.

Parameter	Rural Macro	Urban Macro	Urban Micro
numgNB	1 gNB	1 gNB	1 gNB
hgNB	35 m	25 m	10 m
hUE	1.5 m	1.5 m	1.5 m
maxdist	320 m	250 m	100 m
mindist	35 m	35 m	10 m
numVehicles	[10–60]	[10–60]	[10–60]
speed	120 km/h	30 km/h	30 km/h

**Table 4:** Simulation parameters.

Parameter	Value	Description
channel	mmWave3gpp	Channel model
frequency	28 GHz	Supported Frequency
bandwith	1 GHz	Bandwidth
numSubbands	72	Number of sub-bands
subbandWidth	13.89 MHz	Width of the sub-band (MHz)
propagation	mmWave3gpp	Propagation model
losCondition	true	Channel conditions
shadowing	true	Fading
enableBuildings	true	Consider obstacles
macScheduler	Round-Robin	Scheduler class
harqEnabled	true	Enable HARQ
harqProcesses	100	HARQ for DL and UL
rlcAmEnabled	true	RLC-AM enabled
packetSize	1446 Bytes	Package/Segment Size
segmentSizeFile	matrix	Matrix (n, m)
segmentDuration	2 s	2 s per segment (video)
adaptation	festive, panda and tobasco2	Adaptation algorithm
buffer	524,288 Bytes	Buffer size
simTime	180 s	Total simulation time

## Abbreviations

The following abbreviations are used in this manuscript:

3GPP	Third Generation Partnership Project
5G	Fifth Generation
5GAA	5G Automotive Association
5G-AN	5G Access Network
5GIC	5G Innovation Centre
A-CPI	Application-Controller Plane Interface
AF	Application Function
AMF	Access and Mobility Management Function
AUSF	Authentication Server Function
CN	Core Network
C-V2X	Cellular Vehicle-to-Everything
D2D	Device-to-Device
DASH	Dynamic Adaptive Streaming over HTTP
D-CPI	Data-Controller Plane Interface
DN	Data Network
eMBB	enhanced Mobile Broadband
eV2X	enhancement Vehicle-to-Everything
gNodeB	5G next generation network Node Base Station
HARQ	Hybrid Automatic Repeat Request
HAS	HTTP Adaptive Steaming
HTTP	Hypertext Transfer Protocol
IoV	Internet of Vehicle
ITS	Intelligent Transport Systems
LTE	Long-Term Evolution
MEC	Mobile Edge Computing
mMTC	massive Machine-Type Communication
mmWave	millimeter Wave communications
NBI	Northbound Interface
ns-3	Network Simulator
OF	OpenFlow
PCF	Policy Control Function
PDCP	Packet Data Convergence Protocol
PDR	Packet Delivery Ratio
P-GW	Packet data network Gateway
QoE	Quality of Experience
QoS	Quality of Service
RMa	Rural Macro
RSU	Road Side Unit
SBI	Southbound Interface
SDIoV	Software Defined Internet of Vehicles
SDN	Software Defined Networking
S-GW	Serving Gateway
SMF	Session Management Function
TTI	Transmission Time Interval
UDM	Unified Data Management
UMa	Urban Macro
UMi	Urban Micro
UPF	User Plane Function
URLLC	Ultra-Reliable and Low Latency Communication
V2I	Vehicle-to-Infrastructure
V2P	Vehicle-to-Pedestrian
V2V	Vehicle-to-Vehicle
V2X	Vehicle-to-Everything
VANETs	Vehicular Ad hoc Networks
